# Mitochondrial Bioenergetics and Cardiac Rehabilitation: Bridging Basic Science and Clinical Practice

**DOI:** 10.3390/jcm14113949

**Published:** 2025-06-03

**Authors:** Angela Dziedzic, Klaudia Marek, Piotr Niebrzydowski, Dominika Szalewska, Patrycja Nowak, Elżbieta Miller

**Affiliations:** 1 Department of General Biochemistry, Faculty of Biology and Environmental Protection, University of Lodz, Pomorska 141/143, 90-236 Lodz, Poland; angela.dziedzic@biol.uni.lodz.pl (A.D.); patrycja.nowak5@edu.uni.lodz.pl (P.N.); 2 Department of Neurological Rehabilitation, Medical University of Lodz, Milionowa 14, 93-113 Lodz, Poland; klaudia.marek@umed.lodz.pl; 3 Clinic of Rehabilitation, University Clinical Center in Gdansk, al. Zwycięstwa 30, 80-219 Gdansk, Poland; pniebrzydowski@uck.gda.pl; 4 Division of Rehabilitation Medicine, Faculty of Health Sciences, Medical University of Gdansk, al. Zwycięstwa 30, 80-219 Gdansk, Poland

**Keywords:** cardiac rehabilitation, exercise, cardiovascular disease, mitochondrial bioenergetics

## Abstract

Cardiovascular diseases (CVDs) are the leading cause of global morbidity and mortality, underscoring the necessity of long-term secondary prevention strategies such as comprehensive cardiac rehabilitation (CR). CR is a clinically validated, cost-effective intervention that mitigates cardiovascular risk, improves functional capacity, and enhances patient prognosis. Emerging evidence emphasizes the pivotal role of mitochondrial bioenergetics in mediating the systemic benefits of exercise-based CR, particularly through mechanisms involving mitochondrial biogenesis, dynamics, and mitophagy. This review synthesizes molecular insights with clinical guidelines by evaluating four national CR guidelines—from Poland, France, the United States, and Portugal—alongside a comprehensive recommendation issued by the European Society of Cardiology (ESC). The analysis focused on key components of CR, including exercise modalities (aerobic, resistance, and high-intensity interval training) and prescription parameters such as frequency, intensity, and duration. Only guidelines fulfilling predefined inclusion criteria with complete and detailed data were included; documents lacking essential information were excluded from the final synthesis.

## 1. Introduction

Cardiovascular diseases (CVDs) persist as the predominant cause of morbidity and mortality globally, despite notable advancements in acute medical and interventional therapies. Consequently, long-term management strategies, particularly those aimed at lifestyle modification and functional recovery, have become integral to secondary prevention efforts. Among these strategies, comprehensive cardiac rehabilitation (CR) has emerged as a critical intervention, demonstrating significant efficacy in reducing the incidence of recurrent cardiovascular events, enhancing quality of life, and improving overall prognosis in patients with CVDs [1]. As articulated by Jegier et al. (2021), CR should not be considered a mere adjunctive therapy; rather, it is a clinically validated, cost-effective, and multidimensional intervention that significantly contributes to cardiovascular risk mitigation and long-term patient outcomes [2]. Despite the robust clinical evidence supporting the efficacy of CR, the underlying physiological mechanisms—particularly at the cellular and molecular levels—warrant further elucidation [3]. Recent research increasingly emphasizes the role of mitochondrial function and bioenergetics in mediating the cardiovascular and systemic benefits associated with exercise-based rehabilitation. Mitochondria, often referred to as the bioenergetic powerhouses of the cell, are pivotal regulators of energy metabolism, redox signaling, and cellular adaptation to oxidative stress. Their dynamic responses to physical exercise not only affect skeletal muscle function but also play a critical role in cardiac metabolism, vascular health, and the regulation of systemic inflammatory processes, all of which are essential to the rehabilitative approach [4,5]. This review aims to synthesize insights from basic mitochondrial biology with clinical rehabilitation medicine and cardiology by investigating how exercise-induced mitochondrial adaptations enhance the efficacy of CR. It explores evidence-based recommendations and guidelines pertaining to CR in different countries and current understanding of mitochondrial biogenesis, dynamics, and mitophagy in exercise physiology and its protective role in mitigating pathophysiological conditions in cardiological patients. By combining molecular and physiological research with clinical results, we aim to create a framework that explains the underlying mechanisms of CR and informs future therapeutic strategies.

## 2. Materials and Methods

Databases and publishers were used to search for CR recommendations and guidelines. The electronic databases PubMed, Medline, and ScienceDirect were searched extensively. A systematic review was conducted manually. The following keyword search was used: CR recommendations. The following information was expected: (1) main recommendations and details of the CR program (e.g., risk factor prevention, patient education, physical activity suggested, safety, health monitoring); (2) types of exercise used in the CR program (resistance, aerobic, HIIT); and (3) frequency, intensity, duration, and details of specific forms of training.

**Figure 1:**
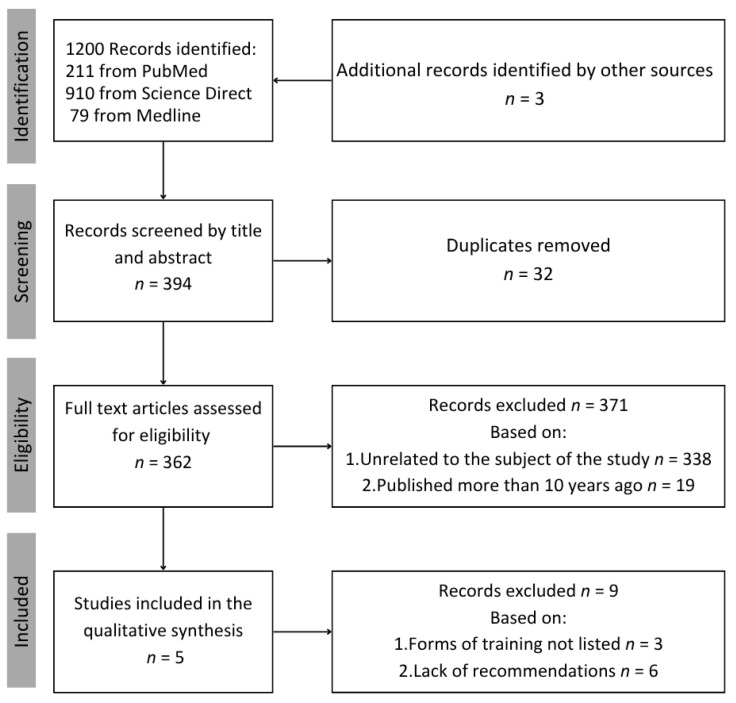
Flowchart of study selection.

In the absence of relevant information (no list of types of training, no main recommendations), the study was excluded due to insufficient data. Five published recommendations and advice on CR were qualified. Four were country-specific: Poland, France, the US, and Portugal. One article was about European recommendations. Four recommendations were published in the English language. One article from Poland was published in Polish. Organizations that provided recommendations and advice were the following: (1) Poland—Section of Cardiac Rehabilitation and Exercise Physiology of the Polish Cardiac Society; (2) France—French Society of Cardiology, Groupe Exercise Rehabilitation Sports—Prevention (GERS-P); (3) the US—American Heart Association (AHA), American Association of Cardiovascular and Pulmonary Rehabilitation (AACVPR); (4) Portugal—Portuguese Society of Cardiology (PSC); and (5) Europe—European Society of Cardiology (ESC). Articles with incomplete data and recommendations were excluded and not considered in the review. Therefore, only five studies that met all requirements were accepted and included in our quality synthesis (Figure 1).

This review considers published recommendations from the past nine years. This systematic review aims to answer the following questions: What are the latest recommendations for CR in different countries?

This review considers published recommendations from the past nine years. This systematic review aims to answer the following questions: What are the latest recommendations for cardiac rehabilitation in different countries?

## 3. Results

CR is a complex process offered to patients who have been diagnosed with heart disease. The intervention includes, but is not limited to, elements of health education, advice on cardiovascular risk factors, dietary advice, physical activity, and stress management [6,7,8]. Evidence from a number of published systematic reviews and meta-analyses suggests that CR reduces mortality, recurrent cardiac events, and hospitalization, and improves exercise capacity, quality of life, and mental health. Recommendations for the use of CR are increasing and it is now recommended in international guidelines [6,9].

Specific guidelines for CR were collected and compared, distinguishing the following aspects (Table 1):

1. Breakdown of rehabilitation into stages/phases
2. Psychological support
3. Training plan
4. Multidisciplinary team
5. Patient education
6. Health monitoring
7. Sexual activity
8. Pharmacotherapy
9. Nutritional counselling
10. Daily living and return to work

**Table 1:** Comparing key factors in CR programs around the world.

Factor/Organization	Cardiac Rehabilitation and Exercise Physiology Section of the Polish Cardiac Society 2021, Poland [2].	French Society of Cardiology, Groupe Exercise Rehabilitation Sports—Prevention, 2023, France [10].	American College of Cardiology, American Heart Association, JACC Expert Panel, 2024, US [11].	Portuguese Society of Cardiology, 2018, Portugal [12].	European Association for Cardiovascular Prevention and Rehabilitation, 2016, Europe [13].
Breakdown of rehabilitation into stages/phases	**Stage I—Early (in-hospital) rehabilitation:** Begins during hospitalization after a cardiovascular event and continues until the patient is discharged from the hospital. Carried out in the intensive care unit, post-operative care unit, cardiology, internal medicine, or CR. **Stage II—Early rehabilitation (outpatient/inpatient):** Can be performed entirely in the inpatient setting, in a center/day unit (outpatient), or as a hybrid cardiac telerehabilitation (HCTR). Inpatient form for patients at high cardiovascular risk, with complications after treatment of acute coronary syndromes (ACSs), cardiac surgery, or percutaneous coronary intervention (PCI), with stable, advanced heart failure (HF) in NYHA class III–IV, immediately after heart transplantation (HT), or for those who, for logistical reasons, cannot participate in rehabilitation programs. **Stage III—Late (outpatient) rehabilitation:** Implemented in a day/outpatient setting Includes health education program for patient and family. Continuous monitoring based on individual needs and cardiac risk profile. Stage III continues for the rest of the patient’s life	No specific breakdown	No specific breakdown	**Hospital Phase I:** begins 24–48 h after a patient is stabilized following an acute event. Includes early mobilization, low-intensity exercise and education. **Early Post-Discharge Phase II:** begins within two weeks of hospital discharge or after diagnosis. It can be conducted in the hospital, a specialized CR center, or in the patient’s home. Includes individualized exercise, education, and lifestyle modification. **Long-term Phase III:** begins after Phase II and continues for the rest of the patient’s life. Aims at long-term maintenance of rehabilitation effects, control of risk factors, and monitoring of health status.	No specific breakdown
Psychological suport	Assessment of mental status and development of an individual psychological care plan. Psychological assistance, especially for sleep disorders, anxiety, depression, mental health deterioration, and reduced quality of life.	Psychosocial assessment at the beginning of the rehabilitation process. Access to psychologist during rehabilitation. Mental status monitoring.	Detailed psychological assessment, including depression, perceived stress, anxiety, sexual dysfunction, anger, loneliness, social isolation, and problematic substance abuse. Psychological interventions and patient education.	Assessment and psychological interventions to reduce stress, anxiety, and depression.	Assessment of psychosocial factors. Specialized psychological interventions.
Training plan	Individualized exercise program for each patient. The patient must be in a stable clinical condition to begin training. Aerobic endurance training involving large muscle groups—3 days per week/day. Resistance training—2 times a week on non-consecutive days of the week Duration of exercise: 20–30 min minimum (45–60 min preferred) per session. Training intensity should be based on individual exercise tolerance and cardiovascular risk. Train according to the FITT rule (frequency, intensity, time-duration, type of exercise). Energy expenditure during exercise of 1000–2000 kcal/week.	Individual exercise program for each patient Frequency: 3–6 times a week Duration: Warm-up: 5–10 min Exercise proper: 20–45 min —Cooling down: at least 5 min. Type: Aerobic Continuous at moderate intensity Interval Resistance. Respiratory: The program should last at least 12 weeks.	Individualized plan, updated every 30 days with health assessment Aerobic training—3–5 days a week, Moderate intensity (40–59%) and high intensity (60–89%) with effort assessment, 20–60 min. Resistance training—2–3 days a week, on non-consecutive days, 10–15 repetitions with 40–60% 1-RM load (maximum load that can be lifted once) with effort assessment.	**Early Phase I**—Early mobilization and low-intensity exercises. Intensity: Determined by the subjective feeling of exertion as assessed by the Borg scale, without exceeding the resting heart rate by 20–30 beats per minute, depending on the patient’s clinical condition. **Phase II** early after hospital discharge. Individualized training program including aerobic and resistance exercises Duration: 8–12 weeks.	Regular physical activity is recommended as a lifelong lifestyle for all men and women, including ≥150 min/week of moderate-intensity activity or ≥75 min/week of vigorous-intensity activity. Aerobic exercise. Resistance training—2–3 sets of 8–12 repetitions at an intensity of 60–80% of 1 repetition performed at a person’s 1-ROM, with a frequency of ≥2 days per week. Neuromotor training.
Multidisciplinary team	Physician, physiotherapist, nurse, radiology technician, psychologist, nutritionist, rehabilitation management specialist.	Physician, nurse, physiotherapist, social worker, nutritionist, psychologist, physical activity trainer.	Physician, nurse, physiotherapist, nutritionist, respiratory therapist, behavioral health expert.	Cardiologist, physiotherapist, rehabilitation nurse, nutritionist, psychologist, psychiatrist, and other specialists as needed by the patient.	Physician, physiotherapist, nurse, nutritionist, pharmacist, sports medicine expert.
Patient education	Risk factor modification: dyslipidemia, hypertension, diabetes, obesity, smoking, and physical inactivity. Healthy lifestyle and physical activity, pharmacotherapy, symptom self-management, and stress management.	Modification of cardiovascular risk factors. Nutrition education. Physical activity education.	Modification of risk factors: physical activity, diet, mental health, sleep, avoidance of unhealthy behaviors (smoking, alcohol, drug abuse).	Counseling on healthy lifestyle, diet, exercise, returning to work, and managing stress and anxiety. Maintaining long-term control of cardiovascular risk factors and adherence to medications and healthy lifestyles.	Disease knowledge. Modification of risk factors—smoking, unhealthy diet, physical inactivity, hypertension, hyperlipidemia, and diabetes. Stress management education.
Health monitoring	Observation of the patient. Assessment of clinical condition before each training—measurement of blood pressure and heart rate. Performing tests: ECG, exercise test, Cardiopulmonary Exercise Test (CPET), 6-min walk test (6-MWT), laboratory tests, transthoracic echocardiography (TTE).	Regular review of risk factors. Monitoring: heart rate and oxygen saturation during exercise. Physical fitness assessment: 6-min walk test, Cardiopulmonary Exercise Test (CPET), ankle–arm index measurement, arterial echo-doppler, Holter-EKG, ambulatory blood pressure monitoring, overnight polygraphy, pulmonary function testing.	Monitor blood pressure and ECG during exercise, especially in high-risk patients. Evaluate progress and adjust exercise plan based on patient response and results achieved.	Risk factor control: Interventions to control hypertension, diabetes, hyperlipidemia, obesity, and smoking. Medical evaluation: Assessment of medical history, risk factors, functional status, and test results (ECG, blood tests, echocardiography).	Monitoring of risk factors: BMI, cholesterol, blood pressure, smoking. Monitoring treatment.
Sexual activity	Information on how to implement physical activity and return to sexual activity is included in patient education.	Therapy related to sexual activity must be offered to both men and women, taking into account the psychological dimension and individual wishes of the patients. Determine the cause of the difficulty: may be due to the disease itself, comorbidities, and medications used (beta-blockers, diuretics, antihypertensives). Possible use of phosphodiesterase inhibitors. Patients with unstable cardiovascular disease or symptoms should not engage in sexual activity until their condition has stabilized.	Psychosocial assessment, including sexual dysfunction. Psychosocial interventions—sex and intimacy education and counseling.	Patient education includes counseling on returning to sexual activity	Possible occurrence of erectile dysfunction due to a cardiovascular event in the future. Modification of risk factors: hypercholesterolemia, hypertension, insulin resistance and diabetes, smoking, obesity, metabolic syndrome, sedentary lifestyle, and depression. Recommend pharmacotherapy.
Pharmacotherapy	Analysis of existing pharmacotherapy with the possibility of its modification depending on clinical condition and in accordance with general recommendations. Pharmacotherapy education.	Monitor drug therapy: Optimize drug therapy, including anticoagulant therapy, and monitor potassium levels and kidney function after each change in therapy.	Evaluate current treatment and modify if necessary. Patient education—importance of adherence to medication recommendations.	Optimization of pharmacotherapy (phase II). Pharmacologic compliance education.	Optimization of pharmacotherapy. Adherence to recommendations. A multicomponent pill (polypill) may be considered to improve adherence to prescribed pharmacotherapy.
Nutritional counselling	Recognize and counteract the effects of malnutrition. Education about healthy nutrition by a nutritionist.	Mediterranean diet. Eating at least 5 servings of fruits and vegetables per day. Limiting the intake of salt and products containing simple sugars. Avoiding processed foods. Nutrition education.	Diet evaluation. Discussion of eating habits. Nutrition education. Individualized nutrition plan.	Dietary counseling, including assessment and advice from dietitians.	Individual dietary recommendations. Avoiding overeating. When following a healthy diet, supplements are not recommended. Limit salt intake. Mediterranean diet. Nutrition education.

Abbreviations: HCTR—Hybrid comprehensive telerehabilitation, ACS—Acute coronary syndrome, HF—Heart failure, HT—Heart transplantation, PCI—Percutaneous coronary intervention, FITT—frequency, intensity, time-duration, type of exercise, CPET—Cardiopulmonary Exercise Test.

According to recommendations from Poland and Portugal, CR is divided into three phases. The first phase is early inpatient rehabilitation, the second phase is outpatient rehabilitation once the patient’s condition has stabilized, and the third phase is late outpatient rehabilitation, which lasts for the rest of the patient’s life. Psychological support for the patient is an important part of the rehabilitation process, especially for patients with adjustment disorders, depression, or anxiety. Psychological support is recommended by all the organizations involved in the review. The detailed presentation of a plan for resistance training, aerobic training, and HIIT is recommended by all countries. The Portuguese Society of Cardiology identified and recommended the above forms of physical activity, but did not elaborate on the intensity, frequency, type, and monitoring of the patient’s health and safety during training.

The need for a multidisciplinary team in the program was raised by all the organizations making recommendations. The team should consist of physicians specialized in physical and rehabilitation medicine (PRM), cardiologists, physiotherapists, nurses, dieticians, psychologists, psychiatrists, radiographers, a pharmacist, and other specialists as required by the patient’s needs. Patient education focuses on elements of risk factor modification, stress management situations, healthy eating, pharmacotherapy, and physical activity. Health monitoring and functional assessment form the basis of a proper CR process. Tests that should be monitored include ECG, blood pressure, echo, stress test, blood tests, and exercise tests. All organizations address the issue of sexual activity after a cardiovascular event. Therapy related to sexual activity must be offered to both men and women, taking into account the psychological dimension and the individual wishes of the patients. The cause of the disorder affecting the patient’s sexual life must be identified. Patients with known unstable cardiovascular disease or symptoms should not engage in sexual activity until their condition has stabilized. If the effects of therapy are not successful, pharmacotherapies should be considered after consultation with a doctor.

All recommendations emphasize evaluation of current treatment and modification with the aim of optimization. Patients should be counseled about medication use and adherence. The European recommendations suggest that a polypill should be considered to improve adherence to prescribed medication in patients with difficulties. Recommendations on dietary advice include an individualized dietary plan for patients, which should be based on the Mediterranean diet. Salt, processed foods, and sweets should be limited and overeating should be avoided [14]. In the US recommendations, there was an emphasis on methods to protect against extreme heat. Extreme heat exposure can be particularly hazardous for cardiac patients. Aerobic endurance exercise is an essential part of a cardiac patient’s training plan [1]. Until recently, resistance exercise was considered unsafe due to the sudden increase in blood pressure and heart rate [15]. Evidence suggests that resistance exercise can be performed safely in cardiac patients up to 90% of 1 repetition maximum (1RM) [16]. The combination of aerobic and resistance exercise has a positive effect on muscular performance and strength [17]. High-intensity interval training, which combines repetitive high-intensity activity with periods of rest or active recovery at lower intensity, is becoming increasingly common in CR recommendations. The increased interest in this type of training is due to positive results in patients with chronic heart disease. In addition, studies have shown improvements in cardiovascular and metabolic function [18]. CR programs in Europe are characterized by low dropout rates among patients and short average wait times for rehabilitation after a myocardial infarction. The state ensures the smooth running of the program through public funding [19]. The approach and recommendations for specific recommended forms of exercise, such as resistance training, aerobic exercise, and high-intensity interval training, were compared [Table 2].

There is a consensus in international recommendations on the frequency of resistance exercise in a cardiac patient’s exercise program. Resistance training should be performed 2–3 days per week, on non-consecutive days, at a frequency of 1RM. The Portuguese Society of Cardiology emphasizes that resistance exercise should be introduced in rehabilitation only from phase II of the process, i.e., after stabilization of the condition and after discharge from the ward. Depending on the source, aerobic endurance exercise should be performed 3–6 times a week, or even daily if there are no medical contraindications and the patient responds well to the exercise. Endurance exercise can last from 5–20 min to 60 min. Aerobic training should be modified by checking the parameters of fatigue, response to exercise, increasing or decreasing the intensity and duration. French recommendations suggest that training should be divided into three elements: warm-up, main part, and recovery, which will help the patient to adapt to the exercise. When recommending high-intensity interval training, the organizations emphasize the need to constantly monitor the patient’s health and rule out contraindications to intense exercise. The Polish recommendation is to start training with 10 s to 4 min of moderate or high intensity, then progress to 1–3 min of low or moderate intensity. This particular form of activity should be decided on a patient-by-patient basis by a medical doctor. American organizations do not recommend extreme high-frequency exercise for heart patients. Exercise intensities should be increased cautiously and gradually, taking into account the patient’s individual capabilities and health status. Given that physical training represents a core aspect of CR, it is essential to gain a comprehensive understanding of the function of mitochondrial respiration during exercise in patients with cardiovascular disease.

## 4. Mitochondria Overview in Cardiology and Cardiac Rehabilitation (CR)

In the cardiovascular system, mitochondria are essential for maintaining myocardial bioenergetics, supporting continuous ATP production required for cardiac contraction and vascular function. Impaired mitochondrial function is linked to heart failure, ischemic injury, and endothelial dysfunction, contributing to vascular aging and atherosclerosis [20,21,22]. Beyond their role in disease, mitochondria are critical for endurance performance, as their content, efficiency, and respiratory capacity correlate with maximal oxygen uptake (VO_2_ max), time-trial performance, and lactate threshold [23,24]. Cardiomyocytes are highly dependent on mitochondrial oxidative phosphorylation for ATP generation. Mitochondria provide cardiomyocytes with a continuous energy supply that is essential for their dependence on repetitive calcium (Ca^2+^)-dependent contractile activity. Consequently, reduced bioenergetic efficiency of the mitochondrial network can directly impair cardiac contraction. When the mitochondrial network is damaged, the delicate balance of Ca^2+^ homeostasis and inflammatory regulation is disrupted, leading to alterations in normal cardiac function, including impaired systolic and diastolic function, arrhythmias, arterial stenosis, and other pathological manifestations [25]. Mitochondria are double-membrane organelles responsible for cellular energy production through oxidative phosphorylation (OXPHOS). They vary in size (0.1–5.0 µm in diameter) and form a highly interconnected reticular network, facilitating efficient energy distribution and adaptation to fluctuating metabolic demands [26]. These organelles contain their own mitochondrial DNA (mtDNA), encoding 37 genes, including 13 essential polypeptides of the electron transport chain (ETC). However, the majority of mitochondrial proteins (approximately 1100) are nuclear-encoded and imported into the mitochondria through specialized translocases [27].

### 4.1. Mitochondria Biogenesis

The primary regulator of this process is peroxisome proliferator-activated receptor gamma coactivator-1α (PGC-1α), which serves as a master transcriptional coactivator governing mitochondrial energy metabolism. Its activation in response to exercise is orchestrated through multiple signaling pathways, including AMP-activated protein kinase (AMPK) and sirtuin deacetylase 1 (SIRT1), both of which are sensitive to cellular energy status, as reflected by ATP and NAD^+^ availability [28]. During exercise, recruitment of muscle myofibril motor units generates an action potential that induces the release of Ca^2+^ ions by the sarcoplasmic reticulum. Subsequently, the increase in cytoplasmic Ca^2+^ stimulates ATP synthesis and the production of ROS in mitochondria. This process promotes the translocation of PGC-1α, which increases gene transcription by interacting with transcription factors and accessory proteins on DNA promoters and regulates the expression of NuGEMPs (nuclear genes encoding mitochondrial proteins). Newly synthesized nuclear-derived proteins are then transported into mitochondria by transferases located in the outer and inner mitochondrial membrane, leading to the activation of the mitochondrial misfolded protein response and mitochondrial biogenesis, promoting their protection and repair [29]. Increased energy consumption during exercise leads also to an elevated AMP and ADP against ATP, triggering AMPK activation via phosphorylation of its catalytic α-subunit (Thr172) by liver kinase B1 (LKB1) and Ca^2+^/calmodulin-dependent protein kinase β (CaMKKβ) [30]. Activated AMPK directly phosphorylates PGC-1α, enhancing its transcriptional activity and stability, while also increasing its expression through PPARGC1A gene induction [31]. Simultaneously, AMPK enhances SIRT1 activity by increasing cellular NAD+ levels, resulting in the deacetylation and modulation of the activity of downstream SIRT1 targets including PGC-1α and the forkhead box O1 (FOXO1) and O3 (FOXO3a) transcription factors [32]. This post-translational modification enhances PGC-1α’s functional capacity, strengthening its interaction with transcription factors NRF 1 and 2 (nuclear respiratory factor 1 and 2) and NFE2L2 (nuclear factor erythroid 2-related factor 2), which orchestrate the transcription of genes critical for mitochondrial biogenesis [33]. NRF1 regulates the expression of genes encoding components of the respiratory chain [cytochrome C oxidase subunit IV (COXIV)], mitochondrial transcription factors [transcription factor A mitochondrial (TFAM), transcription factor B1 mitochondrial (TFB1M), and transcription factor B2 mitochondrial (TFB2M)], and proteins engaged in mitochondrial trans-membrane transport [translocase of the inner membrane (TIM) and translocase of the outer membrane (TOM) complexes] [34,35,36], while NFE2L2 regulates mitochondrial biogenesis and protects against oxidative stress by (1) enhancing NRF1 expression, promoting mitochondrial gene transcription for OXPHOS and protein import, and (2) directly activating redox-related genes, such as heme oxyenase-1 (HO-1) and superoxide dismutase 1 (SOD1) [37,38].

### 4.2. Mitochondria Fusion and Fission

In response to exercise or other endogenous and exogenous stimuli, mitochondria dynamically alter their morphology and function through biogenesis, mitophagy, fission, and fusion. Each of these processes is tightly regulated by specific molecular mechanisms, ensuring mitochondrial adaptability and homeostasis [39]. These processes are tightly regulated by a set of GTPase proteins, including mitofusin 1 (Mfn1), mitofusin 2 (Mfn2), optic atrophy 1 (OPA1), and fission 1 (Fis1), dynamin-related protein 1 (Drp1). Fusion, mediated by Mfn1, Mfn2, and OPA1, promotes the merging of mitochondria, allowing for content mixing (enabling the exchange of mtDNA, proteins, and metabolites), efficient ATP production, and OXPHOS optimization. In contrast, fission, regulated by Drp1 and Fis1, facilitates the removal of damaged mitochondria via mitophagy, redistribution of mitochondria during cell division, and metabolic adaptation to stress [40]. One of the crucial proteins in fission is also PTEN-induced kinase 1 (PINK1), which, by accumulating on the outer mitochondrial membrane in response to mitochondrial damage and phosphorylates Drp1 (Ser616), promotes mitochondrial division and interacts with Fis1 to stabilize this process [41]. The balance between these opposing processes is essential for mitochondrial homeostasis [42].

### 4.3. Mitophagy

Mitophagy is a selective autophagic process that removes damaged or dysfunctional mitochondria, ensuring mitochondrial quality control and cellular homeostasis. It is initiated when a loss of mitochondrial membrane potential (ΔΨm) leads to PINK1 accumulation on the outer mitochondrial membrane, triggering the recruitment and activation of Parkin, an E3 ubiquitin ligase. Parkin ubiquitinates mitochondrial proteins such as Mfn1/Mfn2, marking the organelle for degradation. Ubiquitinated mitochondria are then recognized by autophagy adaptors, such as p62/SQSTM1 (sequestosome 1), nuclear dot protein 52kDA (NDP52), and optineurin (OPTN), leading to the formation of an autophagosome. The autophagosome then fuses with a lysosome, forming an autolysosome, where mitochondrial components are degraded and recycled [43].

### 4.4. Effects of Physical Exercise on Mitochondria Biogenesis and Functionality in Cardiological Patients

Cardiovascular diseases (CVDs) remain the leading cause of morbidity and mortality worldwide. CVDs encompass a broad spectrum of conditions characterized by impaired cardiac and vascular function, often driven by metabolic dysregulation, oxidative stress, and chronic inflammation, which are closely linked to mitochondrial dysfunction [44]. These pathophysiological processes contribute to progressive tissue damage, including apoptosis, fibrosis, and remodeling of myocardium and vasculature, which ultimately compromise cardiovascular performance. Exercise training helps counteract these effects by reducing cell death, inflammation, and fibrosis of myocardium, while promoting vascular regeneration through improved mitochondrial redox balance [45]. Regular physical activity and exercise play a crucial role in postponing the development of CVDs. One key factor in the progression of CVDs is the disruption of mitochondrial homeostasis. Exercise training effectively slows both the onset and advancement of these diseases by restoring mitochondrial balance. This involves enhancing mitochondrial biogenesis, promoting fusion while reducing fission, stimulating mitophagy, and preserving mitochondrial structure and function [46]. These conditions are also characterized by a significant regulation of mitochondria-derived intracellular signaling, including reactive oxygen species (ROS) and Ca^2+^ homeostasis [47].

**Figure 2:**
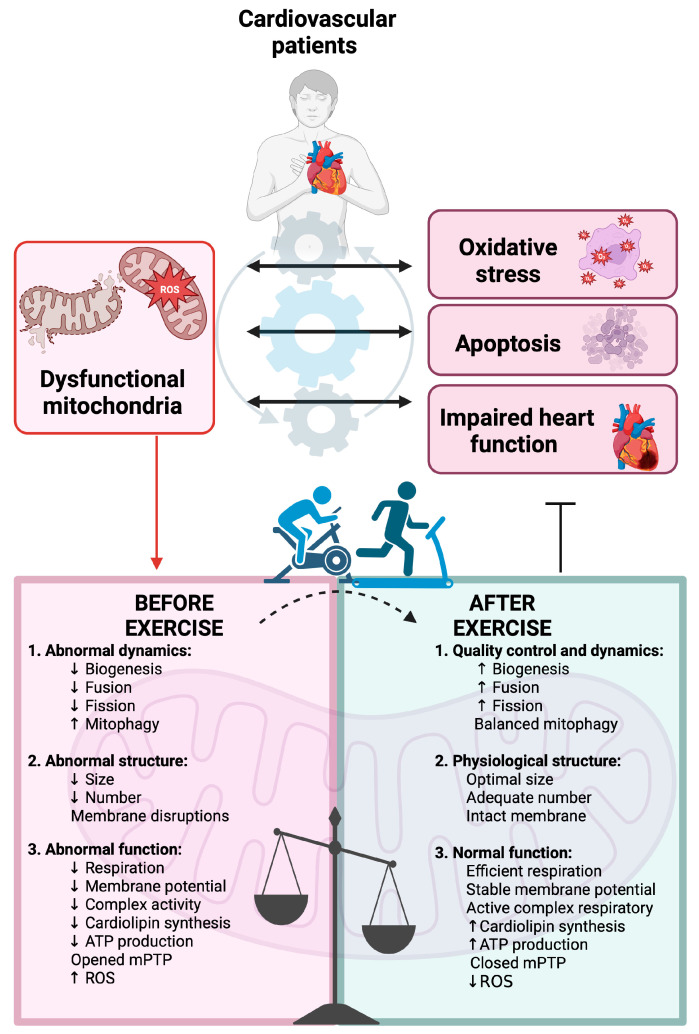
Exercise-induced mitochondrial restoration in cardiovascular (CVD) patients. The role of mitochondrial dysfunction in CVD progression and the beneficial effects of exercise. Damaged mitochondria contribute to oxidative stress, apoptosis, and impaired cardiac function. Regular physical activity restores mitochondrial biogenesis, dynamics, structure, and function, reducing cellular stress and supporting heart performance. Abbreviations: ATP—adenosine triphosphate; mPTP—mitochondrial permeability transition pore; ROS—reactive oxygen species. Created in BioRender. Dziedzic, A. (2025), https://BioRender.com/hte5bak (accessed on 29 May 2025).

The progressive decline in mitochondrial function in CVDs is linked to respiratory chain alterations and impaired ATP synthesis, leading to cardiomyocyte damage and death. This occurs through apoptosis, triggered by cytochrome c release, or necrosis, induced by mitochondrial permeability transition pore (PTP) opening [48]. Inhibiting mPTP is widely recognized as a strategy to reduce cardiomyocyte loss, which contributes to conditions such as myocardial ischemia-reperfusion injury, various cardiomyopathies, and cardiotoxicity [49]. While large, randomized studies on mitochondrial-targeted therapies in CVDs are lacking, numerous preclinical studies highlight mitochondria as a promising therapeutic target. Given their crucial role in energy production and oxidative stress regulation, especially in non-dividing, high-energy-demand cells like cardiomyocytes, maintaining mitochondrial integrity is essential. Coordinating quality control mechanisms including mitochondrial biogenesis, dynamics, and mitophagy, may help prevent mitochondrial dysfunction and slow CVD progression [50,51,52]. All necessary influences of exercise on mitochondrial biogenesis and function in CVD patients are present on Figure 2.

Mitochondrial biogenesis and functional adaptation to exercise are key determinants of aerobic capacity and cardiovascular resilience. Long-term activation of AMPK through regular physical exercise induces important metabolic changes, such as greater mitochondrial sensitivity and optimization of energy metabolism [53]. Physical exercise is a key factor that stimulates mitochondrial biogenesis, which is fundamental in efficient cardiomyocyte metabolism and optimizing cardiac muscle function [54,55,56]. Exercise increases the AMP:ATP ratio, leading to allosteric activation of AMPK, which triggers multiple signaling pathways, including those involved in mitochondrial biogenesis. Regular exercise activates AMPK, enhancing mitochondrial fission and fusion. Campos et al.’s in vivo study demonstrated that a single 4-h swim triggered mitochondrial fragmentation, followed by fusion during recovery, while long-term swimming delayed mitochondrial fragmentation [57]. Mice lacking AMPK β1/β2 isoforms in skeletal muscle show severe physical inactivity and reduced treadmill endurance, linked to lower mitochondrial content [58]. Similarly, AMPKα1α2 knockout mice exhibit weakened exercise capacity, decreased maximal force production, and fatigue resistance, primarily due to complex I dysfunction [59]. Ju et al. report that eight weeks of regular swimming improved mitochondrial biogenesis in skeletal muscle of mice by increasing the expression of PGC-1α and key markers of mitochondrial biogenesis, such as succinate dehydrogenase and cytochrome c oxidase-IV [60]. Likewise, an 8-week moderate-intensity treadmill training program reduced excessive mitochondrial fission and inflammation in the soleus muscle of diabetic rats [61]. Exercise modulates exerkines (musclin, follistatin, and myonectin), which enhance metabolism, inflammation control, and cardiovascular function, helping to reduce cardiac fibrosis, remodeling, and dysfunction after ischemia [62]. Four weeks of treadmill training improved mitochondrial integrity and biogenesis, reducing myocardial damage via SIRT1/PGC-1α/PI3K/Akt activation [63]. In MI mice, eight weeks of swimming (15 min/day) lowered two key mitohphagy markers—microtubule-associated protein 1 light chain 3-II (LC3-II) and p62 levels, while increasing PINK/Parkin expression, enhancing mitophagy. Moderate training elevated SIRT3 levels, reducing ROS, apoptosis, and fibrosis under hypoxic conditions [64].

**Figure 3:**
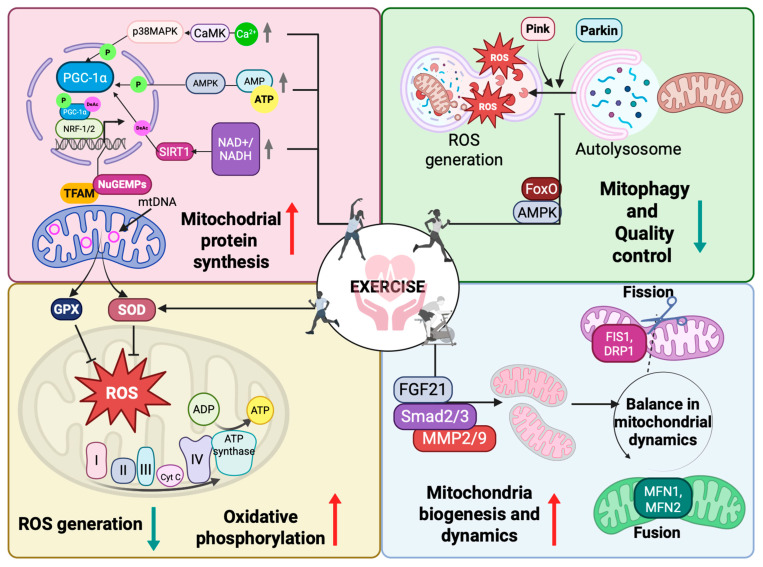
Molecular mechanisms and signaling pathways underlying exercise-induced mitochondrial adaptations. In the upper left panel, exercise activates key regulators such as AMPK, CaMK, and p38MAPK, leading to the upregulation of PGC-1α and downstream transcription factors (NRF-1/2, TFAM), thereby promoting mitochondrial protein synthesis and mtDNA transcription. SIRT1 activation via NAD^+^ also supports this process. In the lower left panel, enhanced antioxidant defenses (e.g., SOD, GPX) and more efficient electron transport chain activity reduce reactive oxygen species (ROS) generation and improve oxidative phosphorylation, leading to increased ATP production. The upper right panel depicts exercise-induced activation of mitophagy and mitochondrial quality control via the Pink1/Parkin pathway. Exercise upregulates AMPK and FoxO signaling, promoting the removal of damaged mitochondria through autophagosome formation and lysosomal degradation. The lower right panel shows improvements in mitochondrial biogenesis and dynamics. Exercise promotes the expression of FGF21 and activation of Smad2/3 and MMP2/9 pathways, restoring the balance between mitochondrial fission (FIS1, DRP1) and fusion (MFN1, MFN2), which is essential for maintaining mitochondrial integrity and function. Abbreviations: AMPK—AMP-activated protein kinase; ATP—adenosine triphosphate; CaMK—calcium/calmodulin-dependent protein kinase; DRP1—dynamin-related protein 1; FGF21—fibroblast growth factor 21; FIS1—fission 1 protein; FoxO—forkhead box O; GPX—glutathione peroxidase; MFN1—mitofusin 1; MFN2—mitofusin 2; MMP2—matrix metalloproteinase 2; MMP9—matrix metalloproteinase 9; mtDNA—mitochondrial DNA; NAD+—nicotinamide adenine dinucleotide; NRF-1/2—nuclear respiratory factor 1 and 2; NuGEMPs—nuclear genome-encoded mitochondrial proteins; PGC-1α—peroxisome proliferator-activated receptor gamma coactivator 1-alpha; Pink1—PTEN-induced kinase 1; Parkin—E3 ubiquitin ligase Parkin; ROS—reactive oxygen species; SIRT1—sirtuin 1; Smad2/3—mothers against decapentaplegic homolog 2 and 3; SOD—superoxide dismutase; TFAM—mitochondrial transcription factor A. Created in BioRender. Dziedzic, A. (2025), https://BioRender.com/bhes4bs (accessed on 29 May 2025).

The few existing human studies align with findings from in vivo research, confirming the same direction of mitochondrial adaptations to exercise. Mendham et al. showed that a 12-week combined aerobic and endurance training program increased the levels of specific intermediate lipids in skeletal muscle, including cardiolipin, phosphatidylcholine, and phosphatidylethanolamine, and enhanced the function of mitochondrial complexes I–V. As a result, respiratory capacity and mitochondrial biogenesis were significantly improved [65]. Endurance training increases mitochondrial volume by 40–50%, leads to higher mitochondrial protein content, improving β-oxidation, and electron transport chain functioning [65]. Mitokines, such as fibroblast growth factor 21 (FGF21) and growth differentiation factor 15 (GDF15), are signaling molecules that mediate mitochondrial communication and stress adaptation. Exercise helps maintain mitochondrial protein homeostasis by regulating mitokine expression. An 8-week endurance training program increased FGF21 levels, exercise capacity, and muscle fiber distribution, while GDF15, a stress-responsive myokine and cardiokine, was elevated during intensive exercise, highlighting its role in exercise-induced adaptations [66]. Porter et al. demonstrated that 12 weeks of resistance exercise training led to both qualitative and quantitative enhancements in skeletal muscle mitochondrial respiration, despite only modest changes in mitochondrial protein content and gene expression. These findings suggest that resistance training effectively improves mitochondrial respiratory capacity and intrinsic function, highlighting its role in optimizing skeletal muscle bioenergetics [23]. The multifaceted impact of exercise on mitochondrial health through molecular signaling pathways that enhance biogenesis, protein synthesis, dynamics, mitophagy, and oxidative phosphorylation are presented on Figure 3.

### 4.5. Non-Invasive Potential Methods for Assessing Mitochondrial Adaptation

Recent studies highlight the importance of assessing exercise effectiveness by identifying biomarkers that reflect functional status and potential mitochondrial adaptations in patients. Various “omics” approaches, such as transcriptomics and proteomics, offer a comprehensive insight into the genes and proteins involved in mitochondrial biogenesis, revealing their dynamic changes in response to high-intensity exercise. One of the newest approaches to assessing this condition is the measurement of mitochondrial microRNAs (mitomiRs) expression [67,68]. Research underlines the crucial role of mitomiRs in regulating biological adaptations of cardiovascular system to physical activity, such as cardiac muscle growth, mitochondrial biogenesis, vascular development, and metabolic regulation [69]. The exploration of mitomiRs as biomarkers of mitochondrial adaptation is still in its early stages, with only a handful of studies available. However, this emerging field holds immense potential, offering a promising avenue for understanding how mitochondria respond to physiological stress and exercise. Research by Carrer et al. has shown that miR-378 plays a key role in regulating mitochondrial energy production by modulating the expression of PGC1-β, at which elevated levels are commonly observed as a physiological response to endurance exercise [70]. Furthermore, miR-494 plays a pivotal role in mitochondrial biogenesis within skeletal muscle [71,72], while also acting as a negative regulator of SIRT3, a key enzyme that fine-tunes mitochondrial energy metabolism in response to exercise and nutritional signals [72,73]. This dual function positions miR-494 as a critical player in balancing mitochondrial adaptation and metabolic flexibility.

Another method worth noting in this field is a single-cell RNA sequencing (scRNA-seq). This technology allows researchers to examine how different cell populations within tissues, such as skeletal muscle or cardiac tissue, respond to physical activity at the transcriptional level. A study utilizing scRNA-seq to explored exercise-induced cardiac adaptations by analyzing mitochondrial gene expression related to fission–fusion balance in heart tissues. By comparing exercised mouse hearts with bulk RNA-seq data, researchers found that exercise modulates mitochondrial fusion and fission processes, which are critical for cardiac adaptation. Specific mitochondrial gene clusters exhibited increased expression post-exercise, suggesting enhanced mitochondrial biogenesis and metabolic flexibility. The study highlighted that maintaining a dynamic balance between mitochondrial fusion and fission is essential for cardiovascular health and the adaptive responses to exercise [74].

Mitochondrial respiration is a fundamental process in cellular metabolism, driving energy conversion through enzymatically regulated reactions that transform substrate-derived energy into ATP. The Seahorse analyzer enables real-time measurement of oxygen consumption in living cells, providing valuable insights into mitochondrial function. This technology allows for the assessment of four key parameters of mitochondrial respiration: basal respiration, ATP-linked respiration, maximal respiration, and proton leak, offering a comprehensive evaluation of mitochondrial efficiency and bioenergetic health. A study by Pribil Pardun et al. investigated mitochondrial adaptation in C2C12 myotubes using electrical pulse stimulation as a model for exercise training. Their findings suggest that mitochondrial bioenergetic profiles improve in response to chronic stimulation, mimicking endurance exercise adaptations [75]. Another promising technique is imaging with MitoTimer, a fluorescent probe distinguishing newly formed mitochondria (green fluorescence) from older ones (red fluorescence) [76], which confirmed enhanced mitochondrial biogenesis in exercised mice, as indicated by a shift toward green fluorescence. In contrast, a high-fat diet promotes red fluorescence, suggesting reduced biogenesis, an effect counteracted by exercise [77].

Looking ahead, future research should prioritize longitudinal human studies directly investigating mitochondrial adaptations to structured CR programs. Such studies are critical to validate preclinical findings and establish clinically relevant mechanisms underlying exercise-induced cardioprotection. In parallel, efforts should focus on the identification and validation of circulating biomarkers, including mitomiRs or other exerkines (signaling molecules released into circulation during and after physical exercise, contributing to systemic adaptations) to monitor mitochondrial function and responsiveness to exercise. These molecular indicators could support personalized exercise prescriptions, stratify patients by responsiveness, and enhance the translational impact of mitochondrial-targeted therapies in cardiovascular care.

## 5. Limitations

The article has some limitations. It discusses CR in the context of European and American populations, omitting data from Asia, Africa, and other regions of the world. A study by Supervia et al. characterizing CR programs around the world indicates the program’s availability in Africa. However, only eight countries in Africa have CR programs available for patients: Algeria, Benin, Kenya, Mauritius, Nigeria, South Africa, Tanzania, and Uganda. In Asian regions, the authors collected data from the Eastern Mediterranean (EMR), South-East Asia (SEAR), and the Western Pacific (WPR). CR programs were available in 12 EMR countries, including Afghanistan, Bahrain, Iran, Kuwait, Pakistan, Lebanon, Qatar, and Saudi Arabia), 6 SEAR countries (Bangladesh, India, Indonesia, Nepal, Sri Lanka, and Thailand), and 11 Western Pacific countries (Brunei Darussalam, China, Malaysia, Japan, the Philippines, Mongolia, Singapore, South Korea, and Taiwan). The African region shows much lower CR availability compared to the EMR, SEAR, and WPR [78]. CR availability by continent is 17% in Africa [79]. There is a lack of guidance on how to implement CR programs in low-resource settings, particularly in Africa [79]. The available Asian–Pacific recommendations offer regional insights into the objectives and prevalence of CR programs [80]. Despite the greater implementation of CR programs in this region, there is a lack of unified recommendations with detailed information on rehabilitation programs, medical personnel, and specific cardiological diseases. These data were not included in the review due to the lack of detailed and standardized information on CR programs in the African, EMR, SEAR, and WPR. Future studies analyzing CR programs should focus on areas with limited financial resources and high continental latitude, which affect access to CR disparities. Future analyses should compare CR programs in the following regions: Europe, America, Africa, Asia, and Australia.

Another limitation of this study is that most of the evidence presented in this section originates from preclinical animal models. While these studies have significantly advanced our understanding of mitochondrial adaptations to exercise, they present several limitations in terms of clinical translation. First, species-specific differences in cardiac metabolism, mitochondrial biogenesis rates, and redox signaling pathways mean that mechanisms observed in rodents may not fully replicate those in humans. Additionally, the exercise protocols used in animal studies (e.g., forced treadmill or swimming) often differ in intensity, duration, and physiological relevance compared to human rehabilitation programs. Moreover, animal models typically lack comorbidities (such as diabetes, hypertension, or medication use) commonly present in cardiovascular patients, limiting the generalizability of findings. Despite strong evidence supporting the crucial role of exercise in mitochondrial function, particularly in cardiovascular health, research remains largely limited to in vivo studies, with a notable lack of human trials. This gap poses a challenge to translating molecular insights into evidence-based clinical recommendations. To address this, future research should prioritize longitudinal human studies that integrate molecular, physiological, and clinical outcomes to validate the therapeutic potential of targeting mitochondrial pathways in CR.

## 6. Conclusions and Future Perspectives

Physical activity is a cornerstone of CR and a critical element of evidence-based secondary prevention strategies recommended by leading cardiology societies. Beyond its well-established clinical benefits, regular exercise induces profound mitochondrial adaptations, including enhanced biogenesis, improved oxidative phosphorylation efficiency, and greater resilience to oxidative stress. These cellular-level changes contribute to improved cardiac function and vascular health by promoting endothelial function, facilitating angiogenesis, and reducing systemic inflammation. Importantly, these biological mechanisms align with clinical guidelines that advocate for structured exercise interventions, such as aerobic and resistance training, tailored to individual patient profiles. These interventions have been shown to reduce the incidence of recurrent cardiovascular events, lower all-cause mortality, and improve quality of life in patients with coronary artery disease, heart failure, and other cardiovascular conditions. Incorporating mitochondrial health as a therapeutic target within these guidelines could further optimize rehabilitation outcomes. Future research should aim to refine exercise prescriptions based on molecular markers of mitochondrial function and explore synergistic strategies combining exercise with pharmacological agents that support mitochondrial integrity. In this context, physical activity not only enhances cellular resilience but also operationalizes the principles of precision medicine in cardiovascular care.

## Figures and Tables

**Table 2:** Recommendations for resistance, aerobic, and high-intensity interval training in cardiac patients.

Author, Year, Country	Resistance Training	Aerobic Training	High-Intensity Interval Training
Jegier, 2021, Poland [2].	From stage II of rehabilitation, after at least one week of well-tolerated and supervised endurance exercise. Frequency of exercise: 2 times per week on non-consecutive days of the week. Exercise intensity: 30–70% 1 repetition maximum (1RM)—the load at which a maximum of 1 full repetition can be performed for the upper extremities and 40–80% 1RM for the lower extremities, with 12–15 repetitions in each set. Type of exercises: Muscle strength training—resistance exercises and general development exercises, including those to improve flexibility, balance, and inspiratory muscle training. Training planning in patients with heart failure (HF): Initially, training intensity should be no more than 30% 1-RM, 5–10 repetitions, 2–3 sessions per week, 1–3 cycles per session. Then gradually increase to 30–40% 1RM, 12–25 repetitions, 2–3 sessions per week, 1 cycle per session. If tolerance is good, loads of 40–60% 1RM, 8–15 repetitions, 2–3 sessions per week with 1 cycle per session can be used. Determine the time relationship between muscle contraction (1–3 s) and muscle rest (e.g., 1:2 ratio). Patients after cardiac surgery: Exercise that may interfere with sternal fusion should be avoided. Upper body stretching exercises can be introduced 6 weeks after surgery. Patients with left ventricular assist devices (VADs): Resistance training, especially of the lower extremity muscles, is indicated. Exercises that are not recommended include running, rowing, crossfit, abdominal exercises, exercises performed with both arms above the head such as weight-lifting, swimming. Heart transplant patients: to increase muscle mass, bone density, and counteract the adverse effects of immunotherapy.	Physical training should be prepared according to the FITT rule. Physical training in CR should provide exercise energy consumption of 1000–2000 kcal/week. Frequency of training: at least 3 days a week, preferably daily. Moderate intensity: 45–59% of peak oxygen uptake (VO_2_ peak), 50–70% of peak load expressed in watt units [W] above first ventilatory threshold (Wpeak), 55–69% of peak HR (HRpeak), 40–59% of heart rate reserve (HR reserve), 4–6 METs (metabolic equivalent of task) or 12–14 points on Borg’s 6–20-point interval-form exercise severity perception scale. Duration of training session: At least 20–30 min, 45–60 min preferred per session. Type of training, type of physical exercise: Aerobic efforts (marching, trotting, cycling, swimming, rowing, dancing), muscle strength training—resistance exercises—and general development exercises, including those that improve flexibility, balance, and exercise the inspiratory muscles. Form of training: Training can be done in interval or continuous form. Exercise models: Different models (A, B, C, or D) of physical exercise can be implemented in each stage of CR, depending on exercise tolerance and risk of cardiovascular events. Continuous endurance exercise (for patients with HF). Patients with severely impaired exercise capacity should start with short, low-intensity bouts of 5–10 min and gradually increase to 30–45–60 min, 3–5 times per week, increasing the intensity over time. Interval endurance exercise (for patients with HF). Periods (10 s to 4 min) of moderate or high intensity exercise (50–100% of peak exercise capacity) are interspersed with periods (1–3 min) of very low intensity exercise or rest. Typically, an exercise session consists of the four phases described above, preceded by 5–10 min of warm-up and followed by 5–10 min of recovery. Heart transplant patients: exercise intensity during aerobic training should be low at first (<50% VO_2_ peak, 10% below anaerobic threshold or <50% of peak workload) and gradually increased.	Acceptable For patients with heart failure (HF), interval endurance exercise involves periods (10 s to 4 min) of moderate- to high-intensity exercise (50–100% of maximal exercise capacity) interspersed with periods (1–3 min) of very low-intensity exercise or rest. Heart transplant (HT) patients: For heart transplant patients, exercise intensity during aerobic training should be low initially (<50% VO_2_ peak, 10% below anaerobic threshold or <50% peak workload) and gradually increased. The decision to incorporate HIIT should always be made on an individual basis, taking into account the patient’s clinical condition, exercise tolerance, and cardiovascular risk. Medical evaluation and appropriate monitoring are required prior to initiating such training.
Bigot, 2024, France [10].	Frequency: resistance training should be performed 2–3 times per week. The maximum load per repetition (1RM) should be assessed. Assessing the strength of large muscle groups is recommended to guide resistance training. Intensity: Resistance training should be performed at an intensity of 30–70% 1RM for the upper body and 40–80% 1RM for the lower body. Initially, 10–15 repetitions per set are recommended, with the possibility of increasing the load as the rehabilitation program progresses. Recent studies suggest that higher intensities (70% 1-RM, 3–10 repetitions) are preferred over more repetitions. Session duration: Training sessions should last at least 20–30 min (preferably 45–60 min). Progression: Progress in resistance training should be monitored using the OMNI-RES scale, on which patients rate perceived load from 0 (very easy) to 10 (very hard).	The training protocol uses continuous and/or interval intensity exercises. Interval training is particularly useful for patients with the lowest fitness levels. Frequency: Should take place at a frequency of 3–6 times per week. Session structure: Each session should include: A warm-up lasting 5–10 min. Main part (exercise period) lasting 20–45 min. Calming (rest period) lasting a minimum of 5 min. Intensity is best determined by load and heart rate at the VT1 ventilation threshold. If VT1 data is not available, intensity should be set at 40–59% of peak VO_2_. You can also use Karvonen’s formula to calculate target heart rate: exercise heart rate = resting heart rate + (heart rate reserve × K). K = 0.6 for patients without chronotropic-negative drugs (beta-blockers). K = 0.8 for patients taking chronotropic-negative drugs. Interval training: combines low-impact aerobics phases with more intense phases that result in lactate production. Intensity should take into account ventilatory thresholds (VT1 and VT2). Intense phases should occur at 70–80% of peak VO_2_, 75–90% of maximum heart rate or a Borg score of 14–16. Session duration: Sessions should last a minimum of 20–30 min and preferably 45–60 min. Progression: Intensity may be modified throughout the program depending on cardiovascular and muscular tolerance, as well as the patient’s psychological progress, guided by the Borg scale.	Intensive phases should take place at 70–80% of peak VO_2_, 75–90% of maximum heart rate, or a Borg score of 14–16. HIIT may be particularly useful for patients with the lowest fitness levels. During HIIT, intensity should take into account ventilation thresholds (VT1 and VT2). The intensity can be modified during the program, depending on cardiovascular and muscular tolerance, as well as the patient’s psychological progress, guided by the Borg scale. Safe for patients following myocardial infarction. Use caution and monitor patients for symptoms. In patients with heart failure (HF), HIIT training has benefits in terms of quality of life (QoL), recovery of autonomy, and reduction in rehospitalizations, with improvements in exercise capacity similar to those seen in patients with reduced ejection fraction. HIIT is effective and safe if the exercises are properly adjusted, the intensity is gradually increased, and the patient is regularly monitored for signs of decompensation.
Brown, US, 2024 [11].	Frequency: 2–3 days a week, on non-consecutive days. Intensity: 10–15 repetitions, 40–60% of maximum load per repetition (1RM). Rating of perceived exertion 11–13 on the Borg scale (6–20) 10–15 repetitions with a load that is 40–60% of your maximum one-repetition effort (1RM), or 11–13 reps on the Borg Scale (6–20). Time: 1–3 sets, 8–10 different exercises targeting major muscle groups. Use equipment that is safe and comfortable for the person (bands, machines). You can use weight machines, free weights, elastic bands or your own body weight. Progression: when the upper limit of repetitions is comfortably reached, increase the load by about 5% or use a resistance band with more tension. During remote training sessions, it is important that the patient masters proper exercise and breathing technique (exhaling during muscle contraction and inhaling during relaxation) to maintain safety. With time constraints at rehabilitation centers, focus on multi-joint exercises (e.g., rowing, chest presses).	Frequency: 3–5 days per week. Stable and able-bodied individuals should be encouraged to do additional exercise at home on days they do not attend CR, aiming to exercise ≥ 5 days per week to meet federal physical activity guidelines. Intensity: Moderate: 40–59% of heart rate reserve. This corresponds to 12 or 13 on the Borg scale (6–20). High: 60–89% of heart rate reserve. This corresponds to 14–17 on the Borg scale (6–20). Exercise intensity should be less than 10 beats/min from the heart rate at which symptoms such as angina, drop or plateau in SBP, SBP > 240 mm Hg, DBP > 110 mm Hg, ECG changes, signs of myocardial ischemia, or other symptoms of exercise intolerance occur. Borg scale: You can use the Borg perceived exertion scale (scale 6–20: moderate 12–13 and vigorous 14–16; scale 0–10: moderate 3–4 and vigorous 5–7) to interpret a patient’s exertion. Duration: 20–60 min, including warm-up and cool-down. Length can be increased by 1–5 min per session until target length is reached. Type: Treadmill, cycling, orbiter, rowing, stair climbing, arm/leg ergometry, stepper and others. It is important to include walking, as it is the main form of physical activity performed throughout the day. Other modalities should also be encouraged, especially those that include upper body exercises, if tolerated. Volume: The volume (frequency × intensity × time) completed per session and each week should be assessed. This can be expressed in calories or MET × min of activity. Progression: increase one element (frequency, intensity, duration, type) at a time. Increase duration by 1–5 min per session until the target duration is reached. Increase intensity after reaching the target duration. Increases in intensity of 5–10% are usually well tolerated.	Safe to use. Extreme high frequency exercise is not recommended for heart patients. Exercise intensity should be carefully and gradually increased, taking into account the patient’s individual abilities and health status. Intensity: short periods of high-intensity exercise and with periods of rest or low-intensity exercise. Perform a graded exercise test to objectively determine peak oxygen uptake and target heart rate ranges and to consider ischemic thresholds, intensities associated with abnormal blood pressure response, deviations, symptoms, or arrhythmias.
Abreu, 2018, Portugal [12].	Strength training is recommended in phases II and III of CR. Before beginning strength training, the patient should undergo a medical evaluation and fitness assessment to identify potential risks and determine the appropriate level of intensity.	In phase II, aerobic training of 20–45 min or 30–60 min (Phase III) is recommended at a frequency of 3–5 times per week (Phase II) or 2–5 times per week (Phase III). Individual training program. Physical fitness assessment, including exercise testing, is mandatory in phases II and III, unless the patient is unable to perform such testing. Contraindications to aerobic exercise, such as unstable coronary artery disease, acute heart failure, severe arrhythmias, and other serious clinical conditions, should be considered.	No specific breakdown.
Piepoli, 2016, Europe [13].	Training should target major muscle groups and include movements involving multiple joints or complex movements. Frequency: Training should occur at a frequency of ≥ 2 days per week. Series and repetitions: For each training session, 2–3 series of 8–12 repetitions are suggested. Intensity: should be 60–80% of the value of 1 repetition performed with the maximum load for a person (1RM; the maximum load that can be taken only once). Elderly and untrained: In elderly and very untrained people, it is suggested to start with 1 series of 10–15 repetitions with an intensity level of 60–70% of 1RM Graduation of intensity: For the elderly and very untrained, it is suggested to start with 1 series of 10–15 repetitions at an intensity level of 60–70% of 1RM1. Gradually increasing the load is key to avoiding injury and overtraining. Warming up before exercise and cooling down after exercise can prevent the occurrence of injuries and adverse cardiac events.	Frequency: physical activity should be used at a frequency of ≥ 3–5 sessions per week, and preferably daily. Duration: We recommend ≥30 min per day for 5 days per week of moderate-intensity PA (i.e., 150 min per week) or 15 min per day for 5 days per week of intense PA (75 min per week), or a combination of both in sessions of ≥10 min each. Shorter exercise sessions (i.e., <10 min) may be appropriate, especially in very untrained individuals. Longer duration exercise, 40 and 60–90 min per day, respectively, has been suggested for lipid control or weight maintenance. Intensity: moderate to high intensity aerobic exercise should be recommended. Examples of aerobic activity: cycling or walking, household chores, gardening, work activities, Nordic walking, hiking, jogging, running, cross-country skiing, rollerblading, rowing, or swimming. Progression: inactive people should start gradually, using light to moderate intensity exercise for short periods of time (even < 10 min), with training sessions spread throughout the week.	In selected stable patients, high-intensity interval training may produce even greater improvements in peak VO_2_. Need to assess clinical status: Prior to initiating any exercise training program, including HIIT, it is necessary to assess clinical and functional status. A comprehensive action sequence diagram has been proposed.

Abbreviations: FITT—frequency, intensity, time-duration, type of exercise; HIIT—High-intensity interval training; VO_2_—peak oxygen uptake; SBP—systolic blood pressure; DBP—diastolic blood pressure; ECG—electrocardiogram; 1RM—One-repetition maximum; HF—Heart failure; HT—Heart transplantation; VT1—first ventilatory threshold; VT2—second ventilatory threshold; MET—metabolic equivalents.
